# Establishing the minimal clinically important difference (MCID) of the Zurich Chronic Middle Ear Inventory (ZCMEI-21) in patients treated for chronic middle ear disease

**DOI:** 10.1007/s00405-020-05819-w

**Published:** 2020-01-27

**Authors:** David Bächinger, Robert Mlynski, Nora M. Weiss

**Affiliations:** 1 Department of Otorhinolaryngology, Head and Neck Surgery, University Hospital Zurich, Zurich, Switzerland; 2 University of Zurich, Zurich, Switzerland; 3 Department of Otorhinolaryngology, Head and Neck Surgery “Otto Körner”, University Medical Center, Doberaner Strasse 137-139, 18057 Rostock, Germany

**Keywords:** Chronic otitis media, HRQoL, COM, MCID, Quality of life

## Abstract

**Aim:**

To determine the minimal clinically important difference (MCID) of the Zurich Chronic Middle Ear Inventory (ZCMEI-21), a questionnaire assessing health-related quality of life (HRQoL) in chronic otitis media (COM).

**Methods:**

In this longitudinal study, 103 patients completed the ZCMEI-21 before and after surgery for chronic otitis media. An anchor-based method including a global rating of change (GRC) was used to assess the MCID of the ZCMEI-21.

**Results:**

A total of 103 patients were included. The mean preoperative and postoperative ZCMEI-21 scores were 28.6 (SD 13.6), and 21.8 (SD 12.8), respectively. The mean change was 6.8 (SD 0.8; *p* < 0.0001). A significant correlation between the ZCMEI-21 scores and the GRC was found (*r* =  − 0.5; *p* < 0.001). Using the anchor-based method, the MCID of the ZCMEI-21 was estimated at 5.3 (SD 12.0).

**Conclusions:**

Knowledge of values indicating a clinically relevant change in patient-reported outcome measures is important when interpreting effects of different treatment modalities. This is the first study assessing the MCID of a questionnaire measuring HRQoL in COM, i.e. the ZCMEI-21. We recommend a MCID of 5 in COM patients undergoing surgical treatment. This information substantially increases the usefulness of the ZCMEI-21 as an outcome measure in COM as changes can be assessed with regard to their clinical meaningfulness.

## Introduction

Health-related quality of life (HRQoL) assessment has become an important outcome in clinical trials as well as in clinical practice [1–3]. HRQoL outcome measures used to determine differences in symptoms over time need to be responsive to the change, e.g. to evaluate the effectiveness of therapies. The responsiveness of an instrument to change reflects the usefulness in clinical trials and has to be considered [4]. Even though questionnaire score differences may exhibit statistical significance, this may not necessarily reflect a meaningful difference in HRQoL, i.e. a clinically important difference. Further, small changes in questionnaire scores ascertaining HRQoL cannot be interpreted as clinically meaningful changes due to the unreliability of individual subjective measurements [5]. Additionally, results may be influenced by the sample size of the study. Therefore, it is important to translate questionnaire score changes into clinically relevant concepts. For this reason, the minimal clinically important difference (MCID) has been introduced [6]. The MCID has been defined as “the smallest difference in score in the domain of interest which patients perceive as beneficial and which would mandate, in the absence of troublesome side effects and excessive cost, a change in the patient's management” [6, 7]. On one hand, the MCID may imply successful treatment and on the other hand, when reporting negative results, may imply therapy failure. The MCID can be determined using different methods, of which anchor-based methods have been described being superior to other methods [8, 9].

The Zurich Chronic Middle Ear Inventory (ZCMEI-21) is a questionnaire available in several languages and assesses specific symptoms and their impact on quality of life in chronic otitis media (COM) [1, 10–14]. The ZCMEI-21 was designed as a disease-specific instrument to assess health-related quality of life in patients suffering from COM [1]. However, the ZCMEI-21′s sensitivity to change and MCID is unknown, which hinders the ZCMEI-21′s interpretations and its usefulness in clinical practice and outcome research [2].

The aim of this study was to determine the responsiveness to change and the MCID of the ZCMEI-21 in patients undergoing surgical therapy of COM.

## Methods

### Study design and patient selection

**Fig. 1:**
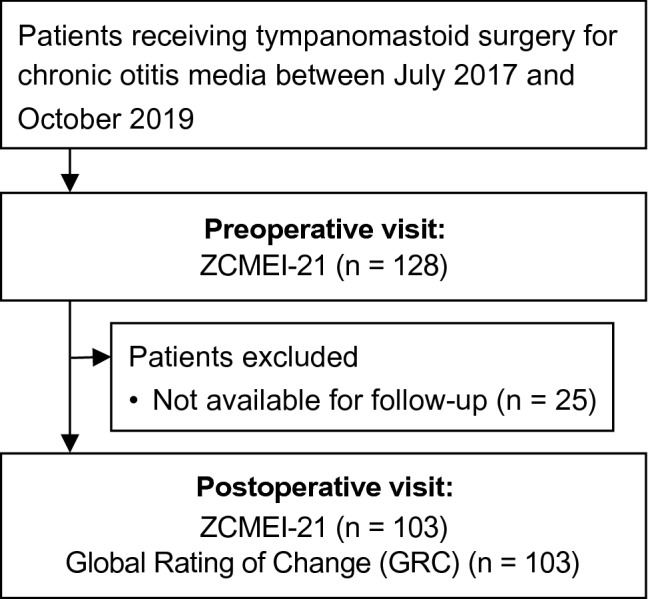
Study flowchart. ZCMEI-21, Zurich Chronic Middle Ear Inventory

In this prospective longitudinal study, patients undergoing surgery for COM were assessed for inclusion (Fig. 1). Patients were recruited from a tertiary hospital, a university medical center (authors RM and NMW). Visit 1 was defined as the preoperative investigation, visit 2 as the postoperative follow-up.

Patients included in the study completed the ZCMEI-21 both at visits 1 and 2. At visit 2, patients additionally completed a questionnaire assessing the global rating of change (GRC) [6]. The MCID was then calculated using an anchor-based method.

### The Zurich Chronic Middle Ear Inventory (ZCMEI-21)

The ZCMEI-21 was used to assess the current HRQoL [1]. The ZCMEI-21 consists of 21 questions grouped into four subscales concerning ear signs and symptoms, hearing function, psychosocial impact and the use of medical resources. Answers are presented using a five-point Likert-scale. High scores correlate with a poorer HRQoL.

### Global rating of change (GRC)

A GRC [6] was used as a patient’s self-assessment of the change in HRQoL of their ear disease between the preoperative baseline visit (visit 1) and the postoperative visit (visit 2). The first question of the GRC assesses whether symptoms improved, remained the same or deteriorated (GRC 1). The second question of the GRC (GRC 2) has a 15-point scoring system with responses ranging from a very great deal better (+ 7) to no change (0) to a very great deal worse (− 7). Scores of 0, − 1 or 1 were considered as indicating no change or an unimportant change. Scores of 2, 3, − 2 or − 3 were considered as indicating a small change, which was considered as equivalent to the minimal important difference. Scores of 4, 5, − 4 or − 5 were considered as indicating a moderate change, and scores of 6, 7, − 6 or − 7 a large change [6, 15].

### Statistical analysis

Statistical analyses were performed using Prism (version 8, GraphPad Software, La Jolla, CA, USA). The significance level was set to *p* < 0.05. The assumption of normality in ABG distributions was assessed using visual inspection of quantile–quantile plots. If not otherwise specified, data are presented as mean with standard deviation (SD) or absolute numbers with percentages.

To assess the responsiveness of the ZCMEI-21, scores between the two visits were analyzed using the paired samples t-test. As an indicator of responsiveness, i.e. the ability to detect any chance, the standardized response mean (SRM) was calculated [4]. Differences in ZCMEI-21 scores among patients grouped according to the GRC 1 were assessed using a one-way ANOVA with post-hoc testing using the Holm-Sidak test. Correlation between the GRC 2 and ZCMEI-21 scores were assessed using Spearman’s rank correlation.

An anchor-based method was used to determine the MCID of the ZCMEI-21. Patients were grouped according to the GRC score bins indicated above. ZCMEI-21 scores from visit 1 were subtracted from visit 2. Thus, negative values indicate an improvement in the HRQoL whereas positive values correspond to a deterioration. Patients whose GRC scores were 2, 3, –2 and –3 were considered to have experienced a small change in their HRQoL. This group was used to determine the MCID of the ZCMEI-21 [6, 16].

## Results

A total of 128 patients undergoing surgery for COM answered the ZCMEI-21 questionnaire preoperatively (visit 1; Fig. 1). Each patient received middle ear surgery within the observed time period. Postoperatively, 103 patients (52 females, 51 males) with a mean age of 51.0 (SD 15.7) completed the ZCMEI-21 questionnaire and the GRC (visit 2) and were included into the study. Indication for tympanomastoid surgery was either primary surgery to eradicate the disease (*n* = 25 COM with cholesteatoma; *n* = 17 COM without cholesteatoma), open mastoid cavity reduction (*n* = 10), revision surgery due to recurrent disease (*n* = 39) or hearing restoration (*n* = 12). The mean follow- up period between surgery and postoperative visit 2 was 183 days (SD 159 days).

### Responsiveness to change of the ZCMEI-21

**Fig. 2:**
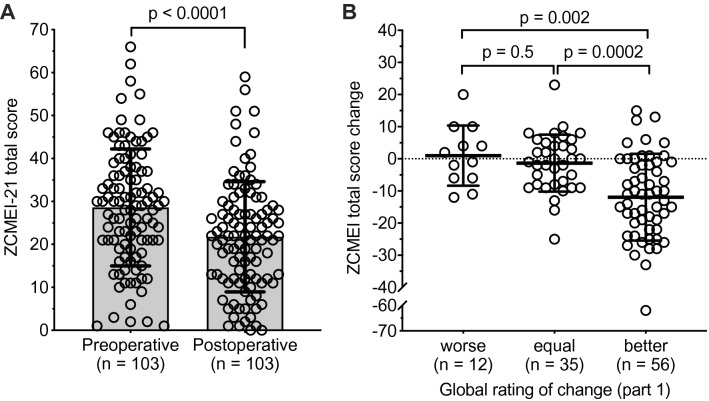
**a** Scatter plot showing ZCMEI-21 total scores of the study cohort before and after surgery. **b** Scatter plot showing ZCMEI-21 total scores in patients grouped according to the first question of the global rating of change. Means are indicated by bars (**a**) or bold lines (**b**). Whiskers indicate standard deviation

The mean ZCMEI-21 score at visit 1 was 28.6 (SD 13.6) and changed to 21.8 (SD 12.8) at visit 2. The mean ZCMEI-21 score change was 6.8 (95% confidence interval 4.3–9.3, SD 0.8, *p* < 0.0001; Fig. 2a). The questionnaire score shifts did not significantly differ between female and male patients (female mean shift = − 6.5 (SD 11.7); male =  − 7.2 (SD 14.1); *p* = 0.76).

The SRM of the ZCMEI-21 was 0.53, indicating a moderate to strong effect size [17, 18].

### MCID of the ZCMEI-21

First, patients were grouped according to the GRC 1. Patients indicating that HRQoL got worse after surgery (*n* = 12), exhibited a mean ZCMEI-21 total score change of 1.0 (SD 9.4; Fig. 2b). Patients indicating that HRQoL was unchanged after surgery (*n* = 35), exhibited a mean ZCMEI-21 total score change of − 1.3 (SD 8.9). Patients indicating that HRQoL was better after surgery (*n* = 56), exhibited a mean ZCMEI-21 total score change of − 12.0 (SD 13.5).

**Fig. 3:**
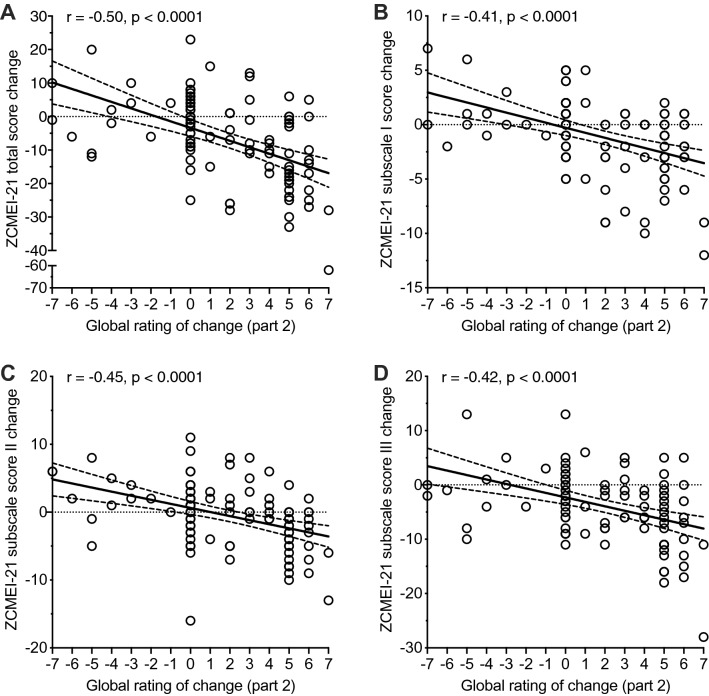
Scatter plot showing ZCMEI-21 scores in patients grouped according to the first question of the global rating of change. **a** ZCMEI-21 total score. **b** ZCMEI-21 subscale score I (signs and symptoms). **c** ZCMEI-21 subscale score II (hearing). **d** ZCMEI-21 subscale score III (psychosocial impact). Solid line represents linear regression line, dashed lines represent 95% prediction intervals. *r*, Spearman’s rank correlation coefficient

A statistically significant correlation was found between the change of the ZCMEI-21 total score and the GRC 2 (*r* =  − 0.5; *p* < 0.001; Fig. 3a). Further, statistically significant correlations were found between the GRC2 and the ZCMEI-21 subscale scores I, II and III (Fig. 3b–d).

Based on the anchor-based method using the GRC (see “Methods” for details), the MCID was calculated within the group that was defined as experiencing a small change after surgery. The mean ZCMEI-21 total score shift in this group was − 5.3 (SD 12.0) and is defined the MCID. The mean ZCMEI-21 scores in patients with ‘no change’, ‘moderate’ and ‘large’ change on the GRCQ were − 1.5, − 11.3, and − 15.4, respectively.

## Discussion

In this study, the MCID of the ZCMEI-21 was determined, thus providing data on which ZCMEI-21 change is clinically meaningful and relevant for patient management and outcome research.

### Main findings

In this study, the MCID of the ZCMEI-21 was estimated to five points. Thus, a change in the ZCMEI-21 total score of an individual patient should only be interpreted as clinically meaningful if it is five points or larger. In accordance, a mean ZCMEI-21 total score should only be interpreted as clinically important if it is five points or larger, even if the score difference is statistically significant. Further, it was found that a fairly linear increase of the ZCMEI-21 total score change with a greater perceived benefit as indicated by the GRC. Patients perceiving no change had a mean ZCMEI-21 total score change around 0 while a small change was associated with a mean ZCMEI-21 total score change around five, i.e. the MCID of the ZCMEI-21. Interestingly, the mean ZCMEI-21 total score change increased in 5-point steps as a moderate change was associated with a ZCMEI-21 total score change around 10 and a large change with a ZCMEI-21 total score change around 15.

This is the first study evaluating the MCID for a questionnaire measuring HRQoL in COM [19]. This sets the ZCMEI-21 apart from other questionnaires measuring HRQoL in COM as the results can now be interpreted with regard to their clinical meaningfulness and treatment modalities. Knowing the value for a clinically significant change of the questionnaire score may assist in patient counselling and adds to the results obtained with the ZCMEI-21 in clinical studies. Therefore, a frequent use of instruments assessing HRQoL in COM may be encouraged both in clinical practice and reseach. Further, ZCMEI-21 total score differences reported in the literature can now be interpreted with respect to the MCID. As an example, Chatzimichalis et al. reported a mean ZCMEI-21-E total score difference of 8.7 (SD 3.3, *p* < 0.01) between unilateral and bilateral COM. Even though this interpretation is hindered by the fact that these data do not originate from the same patients, the results indicate that the difference in HRQoL is clinically important.

### Limitations

Our study has several limitations. First, even anchor-based methods are the preferred method to determine the MCID [8, 9], they depend on the change indicated as important by the patients. This procedure therefore may neglect individual patient’s data. Further analyses could focus on identifying patient subgroups by taken further contributors to the MCID into account, such as gender differences or the severity of the disease [9]. Yet, we consider these contributors as minor in our cohort, as we found no significant differences in the ZCMEI-21 total score changes between male and female patients. Further, within patients suffering from COM, we only selected patients that underwent surgical treatment, which may render the cohort more homogenous.

In this study, the MCID was ascertained in the German version of the ZCMEI-21. Nonetheless, since the ZCMEI-21 was validated in other languages using robust and well-established methods [10, 12, 20], 5 points can be considered to be an appropriate estimate for the MCID of the translated versions of the ZCMEI-21. However, the MCID should ideally be specifically established for each translation.

### Conclusion

In conclusion, this study established an estimate of the MCID of the ZCMEI-21 for COM patients undergoing surgical treatment. Knowledge of the MCID of the ZCMEI-21 may substantially assist in the interpretation of ZCMEI-21 score changes in therapeutic interventions for COM in clinical practice and research. Evaluating whether changes in HRQoL in COM are clinically meaningful increases the usefulness and utility of the ZCMEI-21.
